# Histological comparison of arterial thrombi in mice and men and the influence of Cl-amidine on thrombus formation

**DOI:** 10.1371/journal.pone.0190728

**Published:** 2018-01-02

**Authors:** Julia Novotny, Sue Chandraratne, Tobias Weinberger, Vanessa Philippi, Konstantin Stark, Andreas Ehrlich, Joachim Pircher, Ildiko Konrad, Paul Oberdieck, Anna Titova, Qendresa Hoti, Irene Schubert, Kyle R. Legate, Nicole Urtz, Michael Lorenz, Jaroslav Pelisek, Steffen Massberg, Marie-Luise von Brühl, Christian Schulz

**Affiliations:** 1 Medizinische Klinik und Poliklinik I, Ludwig-Maximilians-Universität, Munich, Germany; 2 Walter-Brendel-Centre of Experimental Medicine, Ludwig-Maximilians-Universität, Munich, Germany; 3 DZHK (German Centre for Cardiovascular Research), partner site Munich Heart Alliance, Munich, Germany; 4 Department of Applied Physics, Center for NanoSciences, Ludwig-Maximilians-Universität, Munich, Germany; 5 Department of Vascular and Endovascular Surgery, Klinikum rechts der Isar der Technischen Universität, Munich, Germany; Hospital for Sick Children, CANADA

## Abstract

**Aims:**

Medical treatment of arterial thrombosis is mainly directed against platelets and coagulation factors, and can lead to bleeding complications. Novel antithrombotic therapies targeting immune cells and neutrophil extracellular traps (NETs) are currently being investigated in animals. We addressed whether immune cell composition of arterial thrombi induced in mouse models of thrombosis resemble those of human patients with acute myocardial infarction (AMI).

**Methods and results:**

In a prospective cohort study of patients suffering from AMI, 81 human arterial thrombi were harvested during percutaneous coronary intervention and subjected to detailed histological analysis. In mice, arterial thrombi were induced using two distinct experimental models, ferric chloride (FeCl_3_) and wire injury of the carotid artery. We found that murine arterial thrombi induced by FeCl_3_ were highly concordant with human coronary thrombi regarding their immune cell composition, with neutrophils being the most abundant cell type, as well as the presence of NETs and coagulation factors. Pharmacological treatment of mice with the protein arginine deiminase (PAD)-inhibitor Cl-amidine abrogated NET formation, reduced arterial thrombosis and limited injury in a model of myocardial infarction.

**Conclusions:**

Neutrophils are a hallmark of arterial thrombi in patients suffering from acute myocardial infarction and in mouse models of arterial thrombosis. Inhibition of PAD could represent an interesting strategy for the treatment of arterial thrombosis to reduce neutrophil-associated tissue damage and improve functional outcome.

## Introduction

Ischemic heart diseases account for 7.0 million deaths per year worldwide [1]. In AMI patients, arterial thrombosis is typically triggered by rupture of an atherosclerotic plaque within coronary arteries [2]. Plaque rupture is associated with disintegration of the endothelial layer and subsequent exposure of the subendothelial extracellular matrix (ECM) [2]. ECM provides a strong activation signal to circulating blood cells and the coagulation system, and triggers a complex series of events that ultimately culminate in the formation of an occluding arterial thrombus. Although the number of deaths due to AMI has decreased [3], efficient strategies to prevent thrombotic complications of coronary atherosclerosis are still lacking.

Platelets are of major importance in the pathophysiology of thrombus formation. They initiate the thrombotic cascade by forming platelet aggregates in a process that depends on platelet integrins, particularly glycoprotein IIb-IIIa [4]. In addition, platelets support the local recruitment of immune cells to nascent thrombi at the site of plaque rupture. The subsequent crosstalk between platelets and recruited immune effectors then results in activation of blood coagulation [5], which propagates thrombus formation and growth in a process termed immunothrombosis [6]. The latter represents a physiological process supported by immune cells and specific thrombosis-related molecules generating an intravascular scaffold that facilitates the recognition, containment and destruction of pathogens, thereby protecting host integrity. However, if uncontrolled, immunothrombosis constitutes the pathophysiologic basis of vessel thrombosis in the absence of pathogens [6]. Monocytes and neutrophils are of particular importance for immunothrombosis, as they deliver tissue factor (TF) and promote TF activation to initiate local coagulation. Further, neutrophils provide extracellular traps (NETs), DNA matrices that act as strong procoagulant surfaces propagating local coagulation [6–8].

Our current knowledge on the cellular and molecular mechanisms underlying arterial thrombosis largely derives from mouse models [9–11]. In mice arterial injury can be induced by several techniques [9], including FeCl_3_ exposure or wire injury. However, it is unknown to date whether these mouse models reflect the phenotype of human coronary thrombi and thus are suitable to obtain data relevant for human pathophysiology. Therefore, we directly compared human coronary artery thrombi of AMI patients to thrombi generated in two distinct mouse models of arterial thrombosis. We found arterial thrombi induced by FeCl_3_ to be highly concordant with human coronary thrombi regarding their cellular composition. We also identified key features of immunothrombosis, specifically neutrophils and NETs, in both human and murine arterial thrombi. Application of Cl-amidine, a pharmacological inhibitor of peptidylarginine deiminase (PAD), into mice reduced arterial thrombosis and myocardial injury. Our findings support recent data on immunothrombosis and suggest that the cross-talk between immune cells and coagulation could represent a target for the treatment of arterial thrombosis.

## Materials and methods

### Retrieval of human thrombi

In this study, we included patients with acute myocardial infarction (AMI) undergoing percutaneous coronary intervention (PCI) that displayed thrombotic occlusion of a coronary artery upon cardiac catheterization. Acute myocardial infarction was defined as ST-segment elevation (STEMI) in the ECG or positive troponin test and/or unstable angina pectoris without ST-segment changes (Non-ST-segment elevation myocardial infarction (NSTEMI)). STEMI was defined as ST-elevation in 2 concordant leads according to current ESC guidelines [12, 13]. In all cases there was an indication for removing the thrombotic material from the coronary artery (cases of stent-thrombosis were not included) according to the guidelines valid during the time of patient enrolment. For this procedure a Pronto^TM^ thrombectomy catheter device (Vascular Solutions, Minneapolis, USA) was used. Approval for this study was obtained from the Ethics Committee of the University of Munich (Project number 4007/11a), and written informed consent was obtained in accordance with the Declaration of Helsinki.

### Definition of thrombus age

We defined thrombus age as period of time between the onset of symptoms according to the patients’ statement and the time point of interventional thrombus removal. In 50 out of 81 patients, the onset of AMI symptoms could be determined precisely, thus allowing the calculation of thrombus age.

Time points of thrombus analysis in mice were driven by the thrombus age calculated in human patients according to their presentation. The mouse model was adapted to match this timing. In 24 out of 50 human patients (approximately 50%) the coronary thrombus was removed within 6 hours after symptom onset. Therefore, we decided to determine the kinetics of leukocyte recruitment after 3 and 6 hours and carried out in-depth histological analysis at those time points.

### Animals

All mice were on C57BL6/J background. Specific pathogen-free mice were obtained from Charles River. All mice used for experiments were between 8 and 14 weeks old and weighed between 21 and 25 g. Each experimental group was weight- and sex-matched so that experiments were carried out on male and female mice with similar weight and in equal distribution between groups. All procedures were performed on anaesthetized animals. Animals were sacrificed by cervical dislocation under deep anaesthesia induced with fentanyl, midazolam and medetomidine. Animal experiments were carried out according to the guidelines from Directive 2010/63/EU of the European Parliament on the protection of animals used for scientific purposes. All experimental procedures on animals met the requirements of the German legislation on protection of animals and were approved by the Government of Bavaria (Regierung von Oberbayern), Germany (reference numbers 55.2-1-54-2532-176-09, 55.2-1-54-2532-182-11 and 55.2-1-54-2532-76-13).

### Assessment of arterial thrombosis after ferric chloride exposure

For thrombus induction in the carotid artery in vivo, we used wire injury (see below) or local application of FeCl_3_ [14]. In brief, mice were anesthetized using 2% isoflurane and intraperitoneal injection of fentanyl (0.05 mg/kg), midazolam (5.0 mg/kg) and medetomidine (0.5 mg/kg). Thereafter, the common carotid artery was exposed. Platelets were either labeled in vivo via intravenous infusion (tail vein catheter) of a fluorescently labeled antibody (X488, DyLight488-labeled, Emfret analytics, Würzburg Germany, 7 μL per mouse in 100 μL sterile PBS), or by infusion of platelets labeled ex vivo with 2’,7’-dichlorofluorescein (DCF). For ex vivo labelling, murine platelets were collected by cardiac puncture and isolated from citrated whole blood as reported previously [15]. Subsequently, platelets were labelled with DCF. After adjustment to a final concentration of 150x10^3^ platelets / 200μl the fluorescently labelled platelet suspension was injected via a tail vein catheter. To induce arterial thrombosis, a filter paper (0.5–1.0 mm) saturated with 10% FeCl_3_ was applied for 3 minutes at the lateral side of the carotid artery adventitial surface (close to the carotid bifurcation) [14]. To investigate leukocyte accumulation over time, thrombus growth was allowed for up to six hours before the vessels were excised.

### Determination of arterial thrombosis after wire-induced arterial denudation

Wire-induced endothelial disruption was performed as described previously [10]. In brief, animals were anesthetized and platelets were isolated from donor mice and labelled with DCF [15]. In recipient mice, the right carotid artery was exposed via a midline neck incision. The common, external and internal carotid arteries were identified and the right internal carotid artery was subsequently ligated with 8–0 silk suture (Ethicon). Additional 8–0 silk ties were looped around the common and external carotid arteries to prevent potential blood loss during the procedure. After transverse arteriotomy of the right internal carotid artery a 0.014-inch flexible angioplasty guide wire was introduced and advanced 10 mm towards the aortic arch. Endothelial denudation injury of the right common carotid artery was performed by passing through the wire three times in a rotating motion to cause endothelial denudation. After removal of the wire, the right internal carotid artery was untied and thrombus growth was allowed for three hours. Subsequently animals were sacrificed and arteries were excised for histological analysis.

### Ischemia-reperfusion injury in mice

Myocardial ischemia reperfusion injury was performed as previously described [16, 17]. In brief, mice were anaesthetized using 2% isoflurane and intraperitoneal injection of fentanyl (0.05 mg/kg), midazolam (5.0 mg/kg) and medetomidine (0.5 mg/kg). Mice were then placed on a heated operating table in a supine position and ventilated using a small animal ventilator (MiniVent, HUGO SACHS, March, Germany) after endotracheal intubation. Access to the heart was gained using a lateral thoracotomy in the second intercostal space. After visualization of the left anterior descending artery (LAD), a suture (8–0 polyamid) was passed underneath the LAD approximately 1 mm distal of the left auricle and tied to a loose double knot. A PE-10 tube was placed between the heart and the knot. The knot was tightened and secured with a second slipknot. Ischemia was confirmed by appearance of a pale color in the myocardium distal to the ligation. After ischemia for 60 minutes, the PE tube and ligation was removed and reperfusion was confirmed by return to a reddish color of the myocardium. After chest closure anesthesia was antagonized using atipamezol (3.75 mg/kg) and flumazenil (0.72 mg/kg), and mice were extubated when spontaneous breathing was sufficient.

### Hemodynamic measurements

Hemodynamic parameters were measured in vivo on day 7 after ischemia. Mice were anaesthetized as described above, fixated on a temperature controlled operating table, intubated and ventilated (MiniVent, Hugo Sachs, March, Germany). A 1.4 French impedance micromanometer catheter (Millar Instruments, Houston, TX, USA) was introduced into the left ventricle via the right carotid artery and pressure volume loops were recorded. Raw conductance volumes were corrected for parallel conductance. Hemodynamic measurements as well as data analyses were performed using PVAN analysis software (Hugo Sachs, March, Germany).

### Infarct size

To evaluate infarct size, mice were euthanized after cardiac catheterization and perfused with 10 ml of 4% formaldehyde solution. Infarct size was determined according to the previous description [18]. In brief, hearts were excised and fixated in 4% formaldehyde solution for 24 hours. Thereafter, they were cut into three 2 mm thick transverse slices (from the base to the apex) and embedded in paraffin. 5 μm thick sections of each slice were cut and mounted on positively charged glass slides and stained using Masson’s trichrome staining. Mean fibrosis area was quantified on transverse slices by a researcher blinded to the treatment groups. Infarct size was determined as area of fibrosis correlated to the area of the left ventricle (including LV-septum).

### Inhibition of NETosis

We used the peptidylarginine deiminase (PAD) inhibitor N-α-benzoyl-N5-(2-chloro-1-iminoethyl)-l-ornithine amide (Cl-amidine, Calbiochem, #506282) to disrupt NET formation [16]. Cl-amidine was dissolved in PBS and a dosage of 10mg/kg was administered. For the study of carotid artery injury Cl-amidine was injected into the tail vein of mice 30 min before ferric chloride exposure [17, 19]. Injection of its vehicle (PBS) served as control. Further, we compared the effect of Cl-amidine on thrombus formation to heparin (100U/kg body weight) injected into the tail vein 10 minutes before ferric chloride exposure [20]. In the myocardial ischemia-reperfusion model Cl-amidine was administered intraperitoneally at onset of ischemia and at time of reperfusion. A third dose of Cl-amidine was administered 12 hours after reperfusion. Vehicle (PBS) injection served as control.

### Intravital epifluorescence microscopy

To maintain a physiological temperature, anesthetized animals were placed on a custom heating mat. The skin of the animals’ neck was opened and the carotid artery was carefully prepared. Stained platelets were injected into the tail vein. Thereafter, thrombus growth in the carotid artery of Cl-amidine and vehicle treated mice was induced using the FeCl_3_ method as described above. In parallel, imaging was started to record the initiation of thrombus formation as well as the duration of arterial occlusion (time from thrombotic vessel occlusion until restoration of blood flow). The time to thrombotic occlusion of the carotid artery downstream of the site of injury was defined as the time required for complete arrest of blood flow in the center of the vessel after removal of the filter paper. While imaging, a prewarmed (37°C) solution of isotonic (0.9%) saline was used to continuously cover exposed regions of the vessel. Measurements were carried out using a Leica DM 6 FS microscope equipped with an Andor Zyla sCMOS camera, or a high-speed widefield Olympus BX51WI fluorescence microscope equipped with a long-distance condenser, a 10x objective with an Olympus MT20 monochromator and an ORCA-ER CCD Camera (Hamamatsu). Leica or Olympus (Cell^R) software were used for image recording and analysis.

### Histology

Human thrombi were harvested during percutaneous coronary intervention as described above. For haematoxylin and eosin (H&E) histology and immunofluorescence stainings, human thrombi were immediately submerged in liquid nitrogen after retrieval and stored at -80°C. Murine carotid arteries were harvested and rinsed with PBS, embedded in O.C.T. compound and frozen at -80°C. Both human thrombi and murine vessels were cut into 5 μm thick sections using a cryotome. Specimens were fixed in 4% formaldehyde solution for 4 min, washed in PBS, and blocked with serum or 5 μg/ml anti-mouse CD16/32 (eBioscience) and 1% BSA (PAA Laboratories) in PBS for 30 min. Sections were incubated with primary antibodies (Table 1) for one hour at room temperature, and then washed in PBS + 0.1% Tween. Detection was performed with fluorescent secondary antibodies (Table 1).

**Table 1:** List of antibodies used for immunohistochemistry.

	Antigen	Primary antibody	Clone	Provider	Secondary antibody	Provider
**mouse**	CD45	Rat	30-F11	eBioscience	Donkey anti-rat Alexa Fluor 488	Invitrogen
	NE	Rabbit	ab68672	Abcam	Goat anti-rabbit Alexa Fluor 594	Invitrogen
	CD68	Rat	FA-11	BioRad	Donkey anti-rat Alexa Fluor 555	Life Technologies
	CD41	Rat	MWReg30	BD	Donkey anti-rat Alexa Fluor 594	Invitrogen
	Fibrinogen	Rabbit	A0080	DAKO	Donkey anti-rabbit Alexa Fluor 594	Invitrogen
	CD45R	Rat	RA3-6B2	BD Pharmingen	Donkey anti-rat Alexa Fluor 555	Life Technologies
	CD3	Hamster	145-2C11	BD Pharmingen	Goat anti-hamster Alexa Fluor 594	Invitrogen
	Ly6G	Rat	1A8	BD Pharmingen	Goat anti-rat Alexa Fluor 555	Invitrogen
	Histone H3	Rabbit	citrulline R2+R8+R17	Abcam	Goat anti-rabbit Alexa Fluor488	Life Technologies
	Tissue factor	Rabbit	Polyclonal	LSBio	Goat anti-rabbit Alexa Fluor488	Life Technologies
	Factor XII	Rabbit	Polyclonal	Novus	Donkey anti-rabbit Alexa Fluor 594	Invitrogen
**human**	CD45	Mouse	2B11+PD7/26	DAKO	Goar anti-mous Alexa Fluor 488	Invitrogen
	NE	Rabbit	ab68672	Abcam	Goat anti-rabbit Alexa Fluor 594	Invitrogen
	CD41	Mouse	P2	Beckman-Coulter	Goat anti-mouse Alexa Fluor 594	Invitrogen
	Fibrinogen	Rabbit	A0080	DAKO	Donkey anti-rabbit Alexa Fluor 594	Invitrogen
	FXII	Mouse	ab1007	Abcam	Donkey anti-mouse Alexa Fluor 488	Invitrogen
	CD20	Mouse	L26	DAKO	biotinylated goat anti-mouse	DAKO
	CD3	Mouse	F7 2.38	DAKO	biotinylated goat anti-mouse	DAKO
	CD14	Mouse	M5E2	BD Pharmingen	Goat anti-mouse Alexa Fluor 488	Thermo Fischer

Leukocytes were identified by using an anti-CD45 antibody in both species. Neutrophils were identified by expression of neutrophil elastase (NE) in humans and mice [7], or using the mouse Ly6G antigen. In mice, monocytes/mononuclear phagocytes were labelled using macrosialin/CD68 [21], whereas in humans we used the marker CD14 [22]. The fibrinogen antibody detected both fibrinogen and fibrin. Murine lymphocytes were identified using CD45R for B-cells and CD3 for T-cells. 1 μg/ml Hoechst 33342 (Invitrogen) was used to stain DNA and Histone H3 antibody was used to visualize histones and NET formation. For control stainings, we used a matching isotype control in combination with the fluorescent secondary antibody. All immunofluorescence images, including controls, were stained with a nuclear dye (DAPI or Hoechst, as indicated).

Human lymphocytes were analyzed by immunohistochemistry. Tissues were stained with antibodies against CD20 to identify B-cells and CD3 to identify T-cells, respectively. Primary antibodies were visualized by Peroxidase/DAB ChemMate Detection Kit (DAKO) according to the manufacturer’s instructions. Samples were mounted using an anti-fade mounting medium (DAKO) and sealed with a coverslip.

### Microscopy and immunofluorescence analysis

Images were acquired using either a Zeiss Imager M2 Axio epifluorescence microscope, or a Leica DMRB epifluorescence microscope with a Zeiss AxioCam and processed with an AxioVision software (Zeiss). Neutrophils and NETs were counted in four fields of view using a 40x objective (176x131 μm). Monocytes, T- and B- lymphocytes were counted in the whole thrombus area. The results were extrapolated to cells/mm^2^ or NETs/100 leukocytes. In our experiments the following prerequisites had to be fulfilled for quantification of NET formation: 1) presence of filamentary structured extracellular DNA, 2) this DNA had to originate from cells stained positively for a neutrophil marker, and 3) respective filamentary structures had to be decorated with a marker for neutrophil granule proteins (like NE) or citrullinated histone H3. To investigate thrombus coverage of coagulation factors (fibrin/fibrinogen, FXII, TF) and platelet area, overview images of the thrombi were acquired and analysed using Zeiss imaging software and ImageJ.

### Statistical analysis

All data are shown as mean ± standard deviation (SD). Baseline characteristics were performed with SPSS. Statistical analyses were carried out using SigmaPlot® 12.0 and GraphPad (Prism 6). To determine differences between groups, data were analysed using a two-tailed unpaired Student´s t-test or one-way ANOVA for multiple comparisons. A value of P<0.05 was considered significant.

## Results

### Phenotype of human coronary artery thrombi

**Fig 1:**
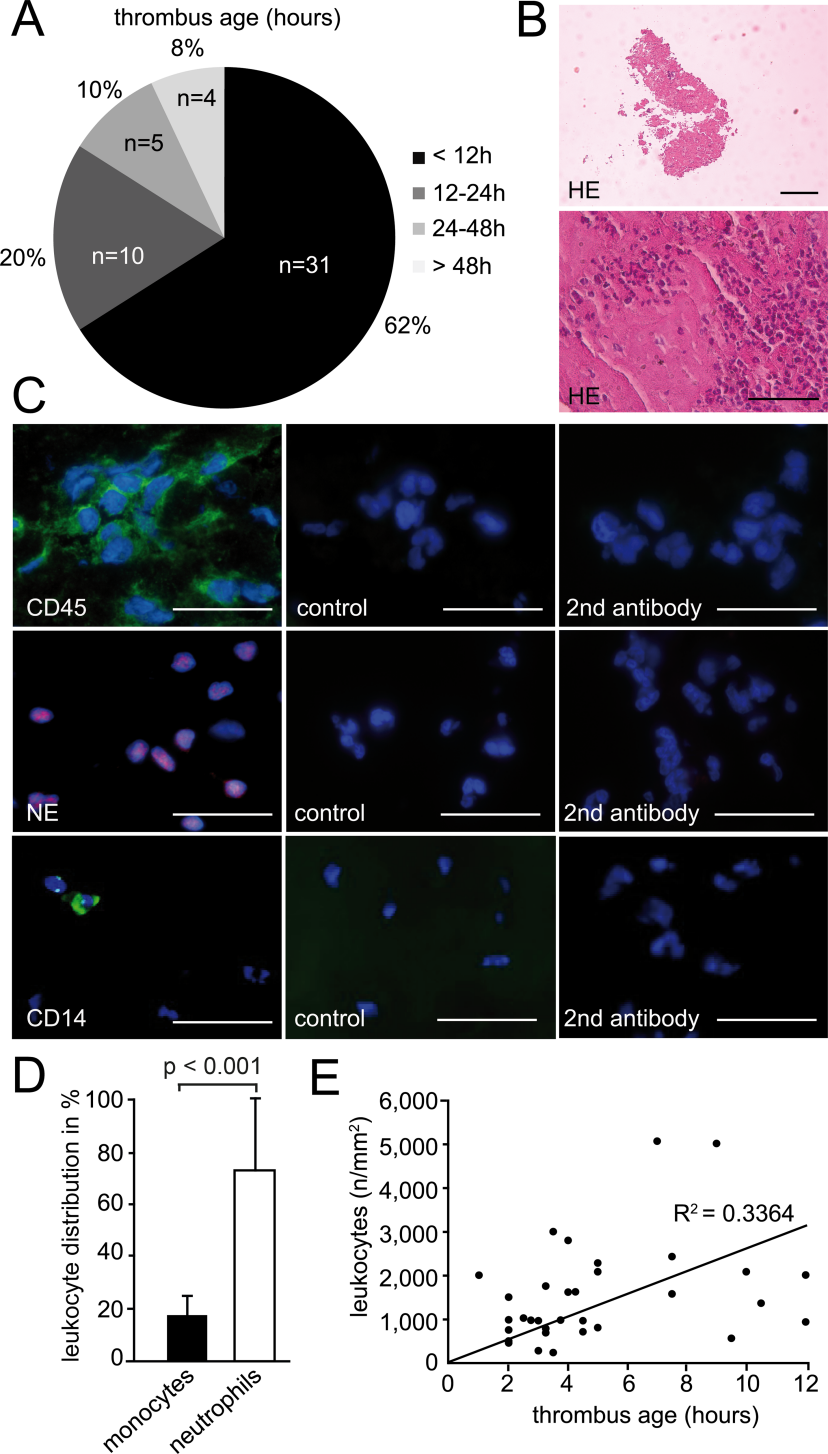
Characteristics of human arterial thrombi. (A) Pie chart shows the distribution of thrombus age. 50 out of 81 patients described the precise onset of AMI symptoms, which allowed the calculation of thrombus age following its removal during PCI. The majority of human thrombi (with precise onset of symptoms) was younger than 24h. (B) Leukocyte accumulation in human thrombi. Representative images of HE staining (n = 3). Bars, 200μm (top image) and 50μm (bottom image). (C) Immunohistochemical visualization of leukocytes (CD45, green, n = 3), neutrophils (NE, red, n = 81) and monocytes (CD14, green, n = 11). Nuclei are counterstained with Hoechst (including controls). Control (isotype) or secondary antibody alone. Bars, 10μm. (D) The graph shows the quantification of monocytes (n = 11) and neutrophils (n = 81) in human thrombi. Results are shown as mean ± SD. (E) Correlation between human thrombi younger than 12h and the number of leukocytes (n = 33).

To evaluate the phenotype of human thrombi in arterial thrombosis we collected specimens of AMI patients undergoing emergency PCI and thrombus aspiration (Table 2). In total, we analysed 81 thrombi of 81 patients (one thrombus per patient). Mean age of the patients enrolled was 62.9 ± 11.2 years. The majority of patients were male (80.2%), presented with STEMI (58%) and one- or three-vessel coronary artery disease (38.3% and 39.5%, respectively). Only few patients had an ejection fraction <30% (5.1%). The most common cardiovascular risk factors were hypercholesterolemia (present in 70% of patients) and hypertension (71.3%). Only 9% of the patients were on dual antiplatelet therapy when admitted to hospital (Table 2). In 50 cases we were able to determine the exact time point of symptom onset, allowing close approximation of thrombus age (for details see materials and methods section). Out of these 50 patients, 62% arrived at the hospital within 12 hours of symptom onset, 20% arrived within 12–24 hours, and 18% arrived later than 24 hours (Fig 1A).

**Table 2:** Patients’ baseline characteristics.

Age (n = 81) ----mean +/-SD	62.9±11.2
Sex (n = 81) ---------n(%)	
Male	65 (80.2)
Coronary artery disease (n = 81) ----n(%)	
1 vessel	31 (38.3)
2 vessel	18 (22.2)
3 vessel	32 (39.5)
Multivessel disease	50 (61.7)
History of coronary bypass	10 (12.3)
History of myocardial infarction	13 (16)
Thrombus origin (n = 81) ----n(%)	
LAD	24 (29.6)
LCx	8 (9.9)
RCA	41 (50.6)
Bypass graft	8 (9.9)
Heart failure (EF < 30%) (n = 79) ----n(%)	4 (5.1)
Risk factors (n = 80) ------n(%)	
Diabetes	16 (20)
Hypertension	57 (71.3)
(Ex)smoker	37 (46.3)
Familial predisposition	33 (41.3)
Hypercholesterolaemia	56 (70)
Presentation at index PCI------n(%)	
Unstable Angina pectoris	4 (4.9)
NSTEMI	29 (35.8)
STEMI	48 (59.3)
Antiplatelet Therapy at ST (n = 78) ----n(%)	
ASA	23 (29.5)
ADP-rec. Antagonist	9 (11.5)
DAPT	7 (9)
Coumarin	1 (1.3)
NOAC	0 (0)
Coexisting conditions (n = 81) ----n(%)	
Renal failure (GFR<30ml/min)	1 (1.2)
Dialysis	0 (0)
Stroke	2 (2.5)
Active Malignancy	2 (2.5)
Atrial Fibrillation	7 (8.6)
Laboratory parameters (n = 81)	
mean +/-SD	
CRP mg/l	18.6±46.4
Leukocytes 10^9/l	11.2±4.6
Platelets 10^9/l	230.3±64.9
CKmax U/L	2111,3±2877.9
CK-MBmax U/l	242.3±319.4
Troponin T max ng/ml	3.3±5.5

In histological and immunohistochemical analyses, human coronary thrombi displayed a heterogeneous morphology with compact, cell-rich regions and areas with fewer cells and less density (Fig 1B). Apart from platelets, leukocytes were present in large numbers in thrombi, supporting a role for inflammatory cells in arterial thrombosis in humans. Thrombus-associated leukocytes were mostly found in clusters or layers (Fig 1C). Further analysis revealed that neutrophils represented the major fraction with 73.4 ± 26%, whereas monocytes constituted 17.9 ± 6% (Fig 1D). Within the first 12 hours after symptom onset, the number of leukocytes increased and correlated moderately with thrombus age (R^2^ = 0.3364) (Fig 1E). Together this suggests that in addition to platelets, neutrophils and monocytes were the most abundant cell subsets that accumulated in thrombi of patients suffering from AMI. This is in line with previous work on thrombi isolated from AMI patients [23, 24].

### FeCl_3_-induced arterial thrombi closely resemble the phenotype of human coronary thrombi

**Fig 2:**
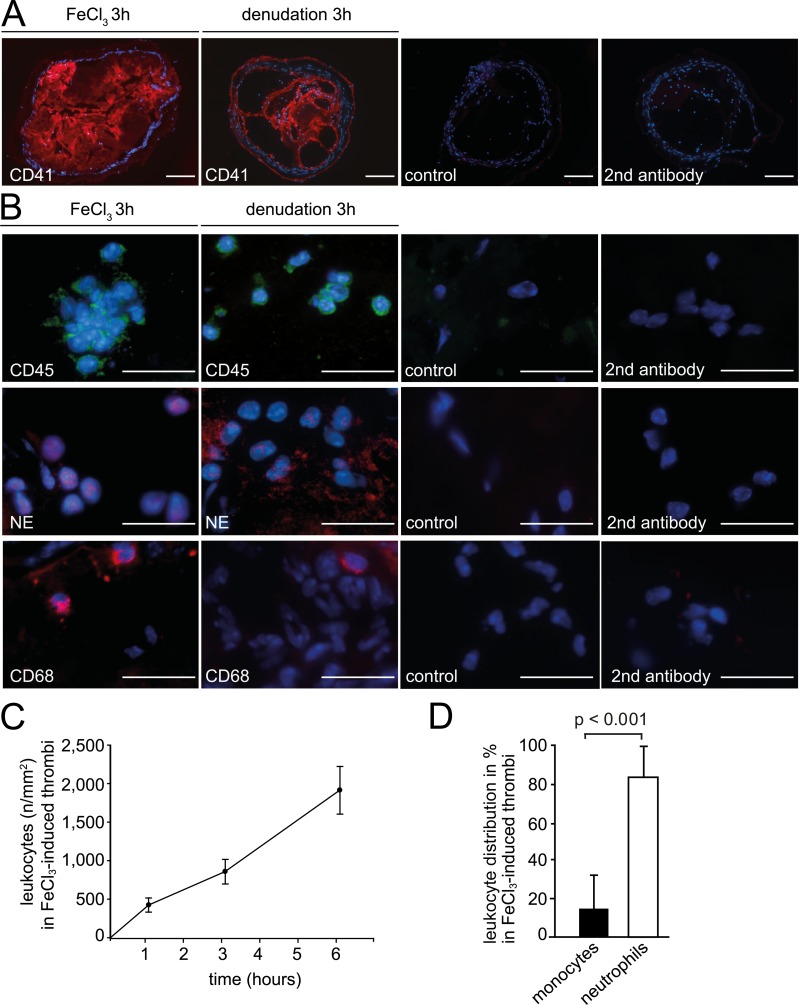
Characteristics of mouse arterial thrombi induced by FeCl_3_ injury or wire denudation in mice. (A) Immunohistological images of platelet aggregate area (red) in arterial thrombi (n = 3/group) and control stainings. Bars, 100μm. Control (isotype) or secondary antibody alone. (B) Comparison of leukocyte recruitment to the mouse carotid artery 3h after FeCl_3_ exposure or wire denudation (n = 3/group). Representative images show immunohistochemical staining for leukocytes (CD45, green) and their subsets, as distinguished by expression of neutrophil elastase (NE, red) for neutrophils and CD68 (red) for blood monocytes. Nuclei were counterstained with Hoechst (including controls). Bars, 10μm. Control (isotype) or secondary antibody alone. (C) Association between number of leukocytes and thrombus age (n = 3/group). Mean ± SD. (D) Quantification of monocyte and neutrophil subsets within mouse thrombi 3h after FeCl_3_ exposure (n = 3/group). Mean ± SD.

As outlined above, various techniques exist to induce arterial thrombosis in mice. In this study we analysed the thrombus phenotype in the two most frequently used models (wire-injury and FeCl_3_) and compared it to arterial thrombi in humans. Similar to humans, CD41+ platelets were a major cellular constituent of mouse thrombi, consistent with their important role in arterial thrombogenesis (Fig 2A, S1 Fig). FeCl_3_ exposure resulted in large, compact thrombi that closely resembled the phenotype of thrombi from AMI patients. In contrast, experimental thrombi obtained with wire denudation were considerably smaller, less dense and did not occlude the entire lumen of the artery. Thus, mechanical injury by wire denudation was a less efficient trigger of thrombosis compared to FeCl_3_, and these thrombi were markedly different to those found in human coronary artery thrombosis (Fig 2A).

**Fig 3:**
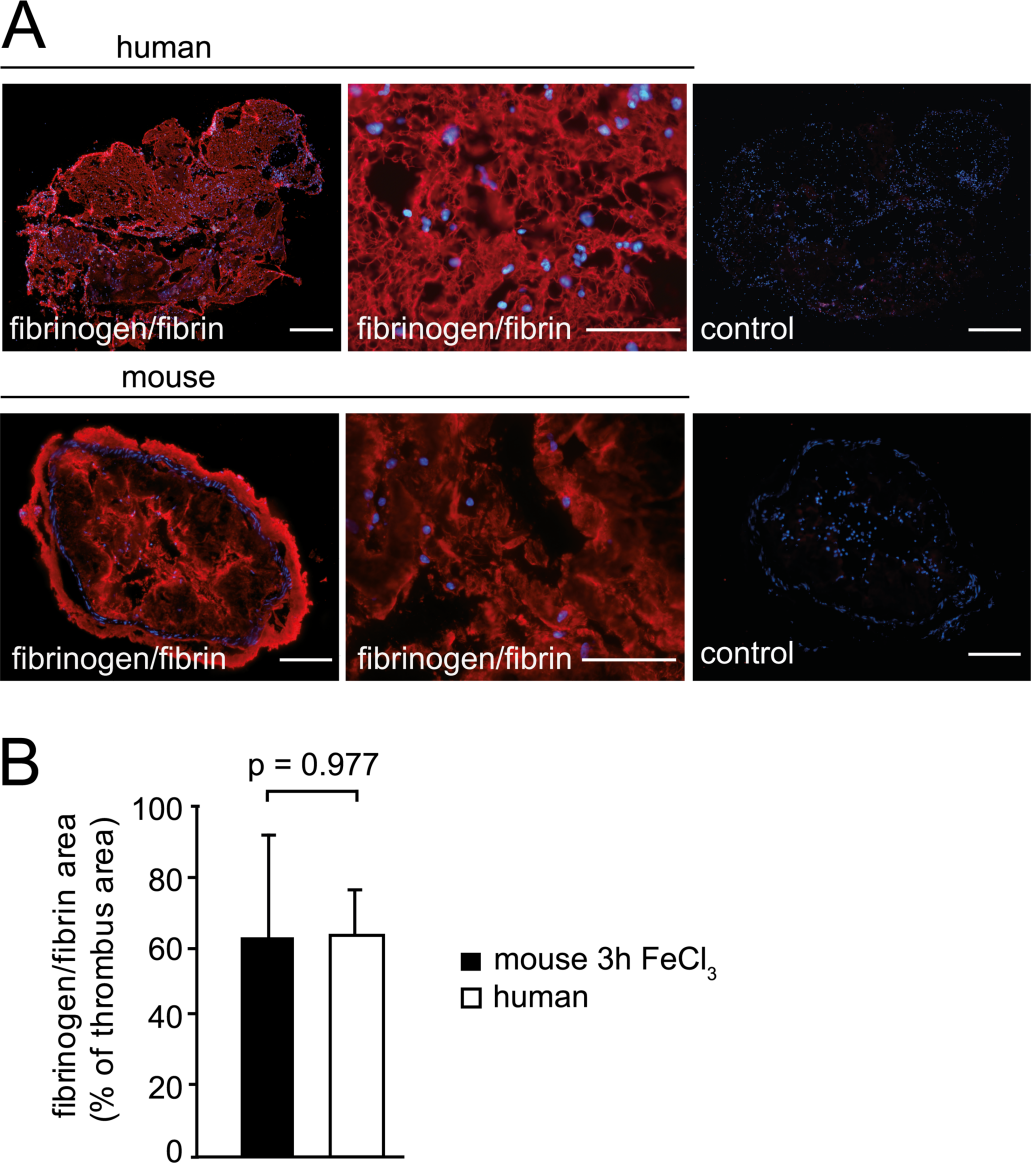
Accumulation of fibrinogen/fibrin in human and mouse arterial thrombi. (A) Representative immunohistochemical staining of mouse and human thrombi for fibrinogen/fibrin (red) and control stainings. Nuclei were counterstained with Hoechst (including controls). Bars: 50μm (top left and right), 200μm (bottom left and right), 300μm (top and bottom middle). (B) Fibrinogen/fibrin-covered area in the thrombus (human thrombi n = 6, mouse thrombi n = 3). Data are shown as mean ± SD.

In addition to platelets, we found large numbers of leukocytes accumulating in murine arterial thrombi. Similar to human thrombi, CD45+ leukocytes in FeCl_3_-induced arterial thrombosis were distributed in clusters or layers (Fig 2B), and showed a substantial increase over time. Because of the close similarities in morphology and cellular composition of thrombus specimen obtained from human coronary arteries and FeCl_3_-treated mouse carotid arteries, we compared these thrombi in more detail. Leukocyte content at different time points of thrombosis and the increase in cell numbers was well comparable between mice and men (Figs 1E and 2C). In thrombi retrieved after 3 hours, we quantified 902 leukocytes/mm^2^ in humans (3h from symptom onset to thrombus retrieval) and 857 leukocytes/mm^2^ in mice (3h after FeCl_3_ application), (Figs 1E and 2C). Further, neutrophils were the predominant leukocyte subset also within mouse arterial thrombi, constituting 81.1 ± 19%, while monocytes accounted for 15.4 ± 8% of leukocytes (Fig 2D). B and T lymphocytes represented only minor leukocyte fractions in both human and murine arterial thrombi (S2 Fig). We next addressed fibrinogen/fibrin (FGN) deposition and found comparable immunofluorescence stainings in mouse and human thrombi (Fig 3A and 3B). Due to the fragmented morphology and smaller size of the thrombi generated in the wire injury model, we were not able to determine the composition of these thrombi in more detail. Taken together, murine thrombi generated by FeCl_3_-exposure closely resembled the phenotype of thrombi harvested from AMI patients containing the major effectors of immunothrombosis, including platelets, fibrinogen/fibrin, and immune cells.

### Presence of NETs in arterial thrombi of mice and men

**Fig 4:**
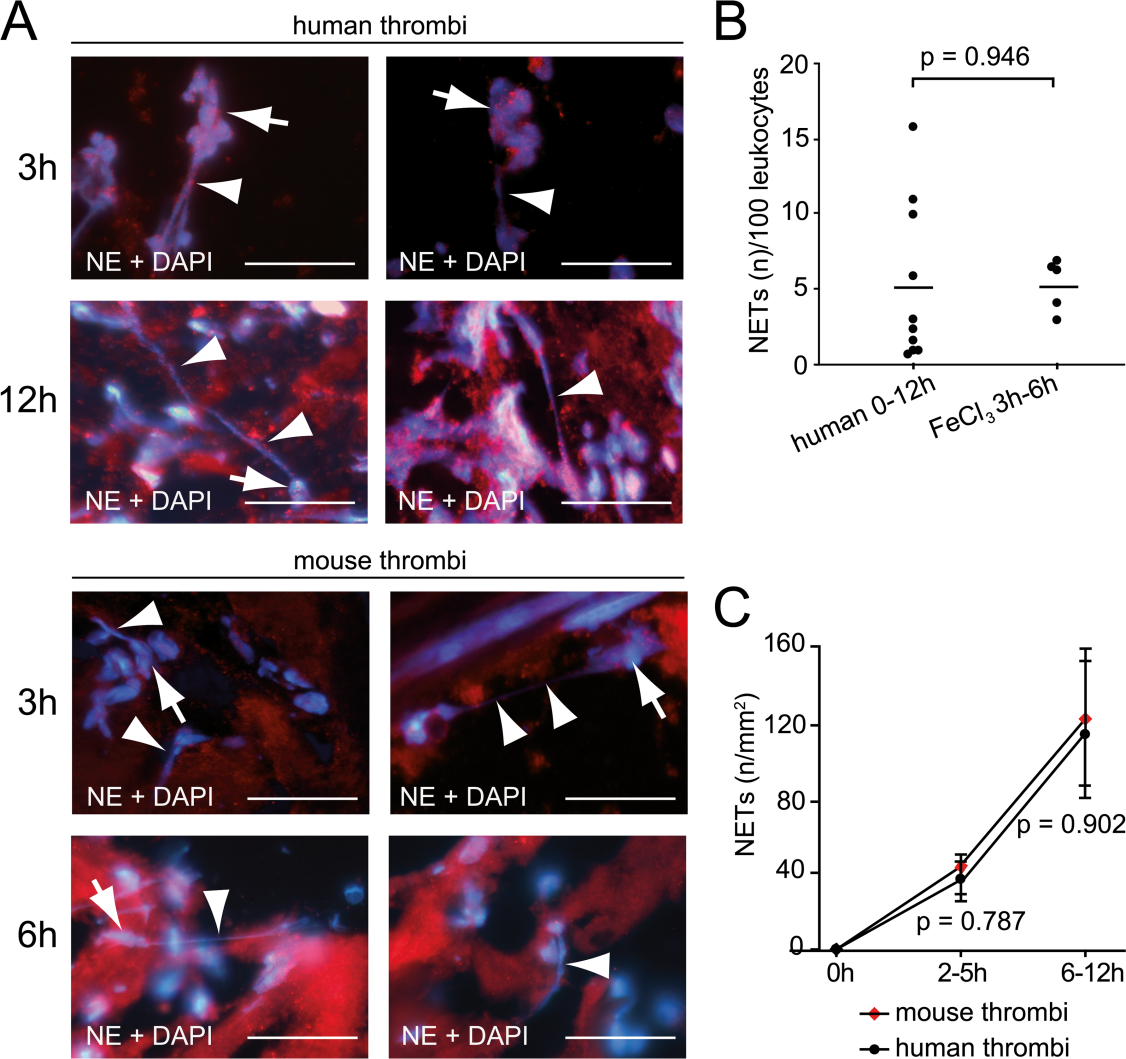
NETs in arterial thrombi of mice and humans. (A) Representative illustration of NETs stained for NE and DNA (DAPI) in the early phase of arterial thrombosis. Human and mouse thrombi showed comparable morphology after 3, 6 or 12h. Extracellular DNA originates from NE+ neutrophils. Bars, 10μm. Arrows, nuclei; arrowheads, NET fibers. (B) Quantification of NETs per 100 neutrophils in human thrombi (<12h) (n = 10) and experimental thrombosis (FeCl_3_) (3–6h) (n = 5). Dots represent individual experiments; lines indicate mean values for each group. (C) Association between thrombus age and number of NETs in mice and humans.

Neutrophils can exhibit strong pro-coagulatory properties by releasing DNA matrices (NETs). In line with previous reports [25, 26], we found extracellular DNA in 23% (19 out of 81) of human coronary thrombi (Fig 4A). Interestingly, the amount of NETs relative to the thrombus leukocyte count was similar between human AMI specimens and FeCl_3_-induced murine arterial thrombi (Fig 4B). Of these 19 patients with NETs, thrombus age could be determined in 10 patients (Fig 4C). The amount of netting neutrophils increased with thrombus age and displayed similar kinetics between mice and men (Fig 4C).

### Cl-amidine inhibits arterial thrombosis

**Fig 5:**
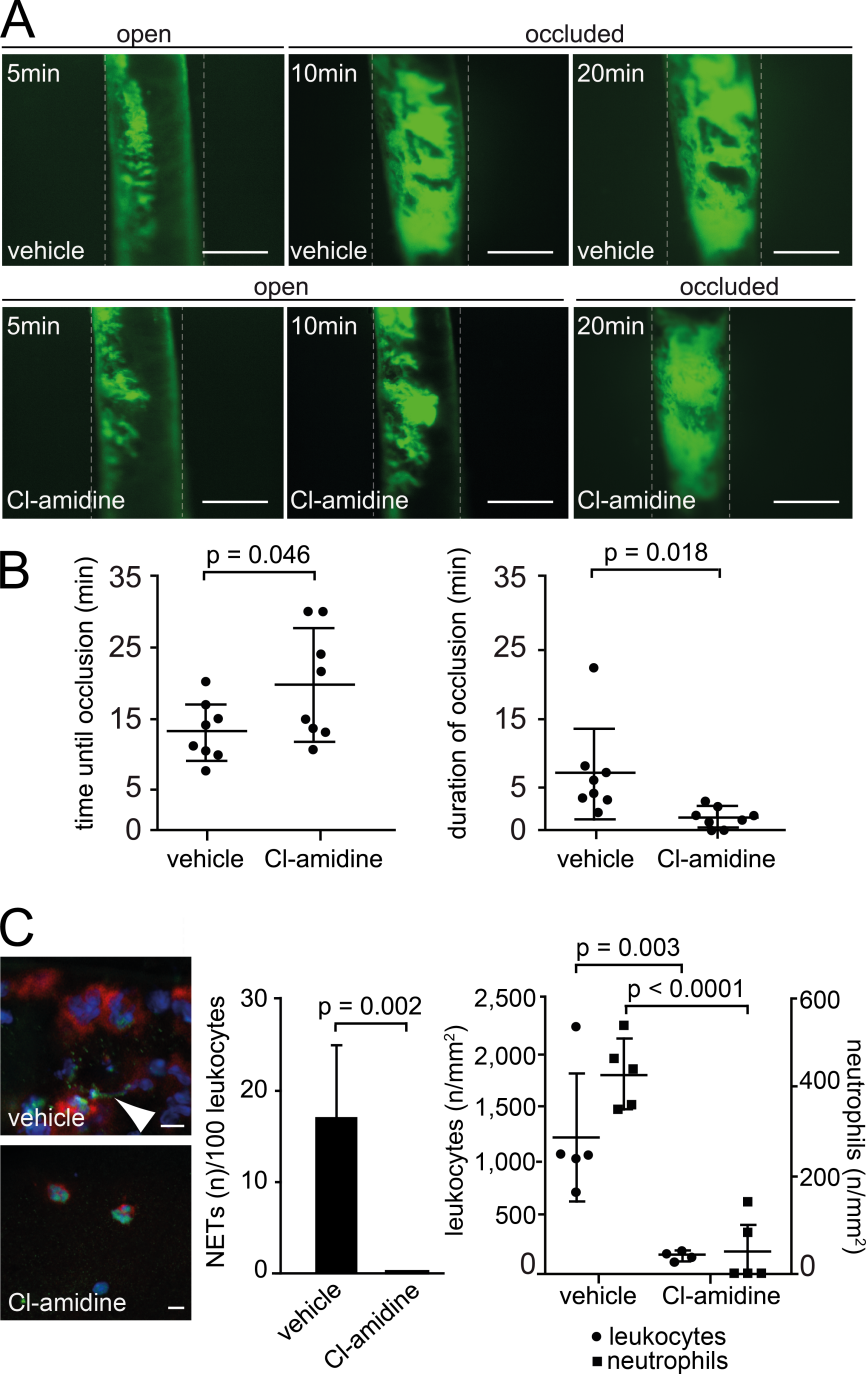
Cl-amidine inhibits arterial thrombosis in mice. (A) Representative intravital microscopy images 5, 10 and 20min after FeCl_3_ injury in mice treated with Cl-amidine or vehicle. Platelets were labeled in vivo (green). Bars, 200μm. (B) Time until occlusion (left) and duration of vessel occlusion (right) after FeCl_3_ exposure in mice treated with vehicle (n = 8) or Cl-amidine (n = 8). (C) Left: Representative histological images (Ly6G in red, cit H3 in green, DAPI in blue) of NETs in mice treated with vehicle or Cl-amidine (n = 5/group). Bars, 5μm. Arrowhead, NET fiber. Middle: Quantification of NETs per 100 neutrophils (n = 5/group). Right: Quantification of leukocytes (left axis) and neutrophils (right axis) in murine arterial thrombi.

A functional role of NETs in thrombosis has previously been demonstrated in atherosclerosis-prone mice [27]. To address the role of NETs in FeCl_3_-induced arterial thrombosis, we visualized thrombus formation using intravital microscopy in the presence and absence of Cl-amidine, which impairs NETosis through inhibition of peptidylarginine deiminase (PAD) [17, 19]. Cl-amidine reduced FeCl_3_-induced arterial thrombosis (Fig 5A and 5B). More specifically, the time until thrombotic occlusion of the carotid artery was prolonged (Cl-amidine 19.9 min ± 7.6 vs. vehicle 13.3 min ± 4.0, n = 8) and re-establishment of blood flow was accelerated (Cl-amidine 1.7 min ± 1.4 vs. vehicle 7.8 min ± 6.3, n = 8) (Fig 5B). Similar results for Cl-amidine were observed by direct labelling of platelets in vivo (Fig 5A) as compared to ex vivo labelling and re-infusion of donor platelets (S3 Fig). Inhibition of thrombus formation by Cl-amidine was robust but not as pronounced as intravenous application of 100U/kg heparin (S4A Fig).

**Fig 6:**
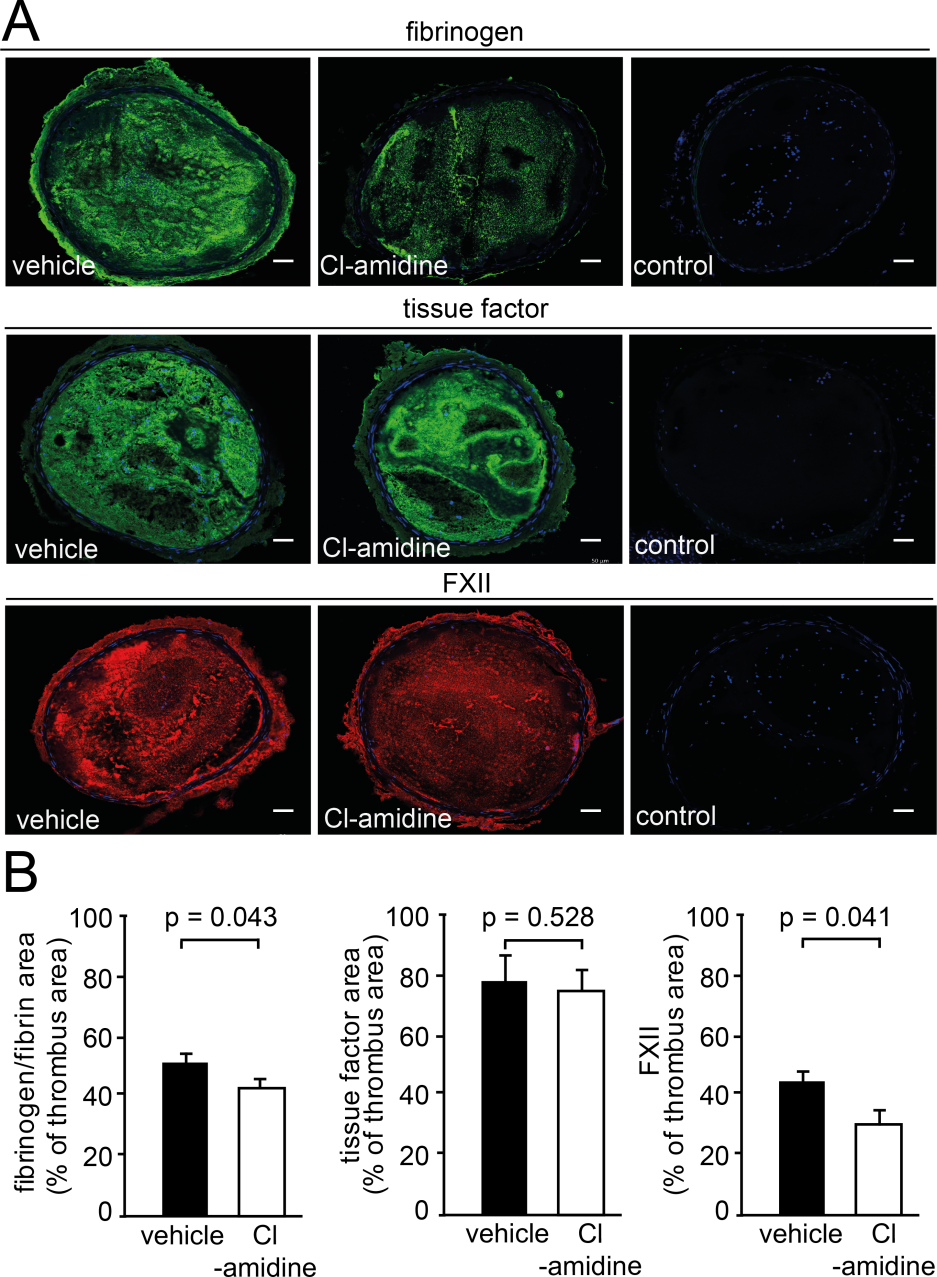
Cl-amidine modulates thrombus composition. (A) Immunofluorescence analysis of fibrinogen (1^st^ row), tissue factor (2^nd^ row) and factor XII (3^rd^ row) in arterial thrombi of mice treated with vehicle or Cl-amidine. Nuclei were counterstained with Hoechst. Controls were stained with isotype and secondary antibody antibody together, and Hoechst, Bars: 50μm. (B) Immunofluorescence staining of coagulation factors in % of whole thrombus area. Left: Fibrinogen-covered thrombus area (vehicle n = 5, Cl-amidine n = 5). Middle: Tissue factor (vehicle n = 5, Cl-amidine n = 4). Right: Factor XII (vehicle n = 6, Cl-amidine n = 5). Results are mean ± SD.

To further address the role of Cl-amidine in thrombus formation, we carried out immunofluorescence stainings of immune cells and coagulation proteins. Unexpectedly, we observed a strong decrease in the number of leukocytes, including neutrophils, within arterial thrombi of Cl-amidine treated mice. Thus, in addition to disrupting NETosis, Cl-amidine depletes leukocytes in arterial thrombi whereas leukocyte numbers in peripheral blood remained stable (S4B Fig). The underlying mechanism remains elusive and will need to be determined in future work. However, absence of distinct leukocyte populations is known to affect thrombus stability [7]. To define the role of Cl-amidine in more detail, we carried out immunofluorescence stainings of key proteins of the coagulation system. Fibrinogen and FXII coverage of the thrombus area was reduced in the presence of Cl-amidine (Fig 6), suggesting that this compound affects not only thrombus composition in respect to NETs and leukocyte content, but also modulates coagulatory properties of the thrombus. Together, these findings support the concept that Cl-amidine could provide an interesting strategy to inhibit arterial thrombosis.

### Cl-amidine reduces myocardial injury

**Fig 7:**
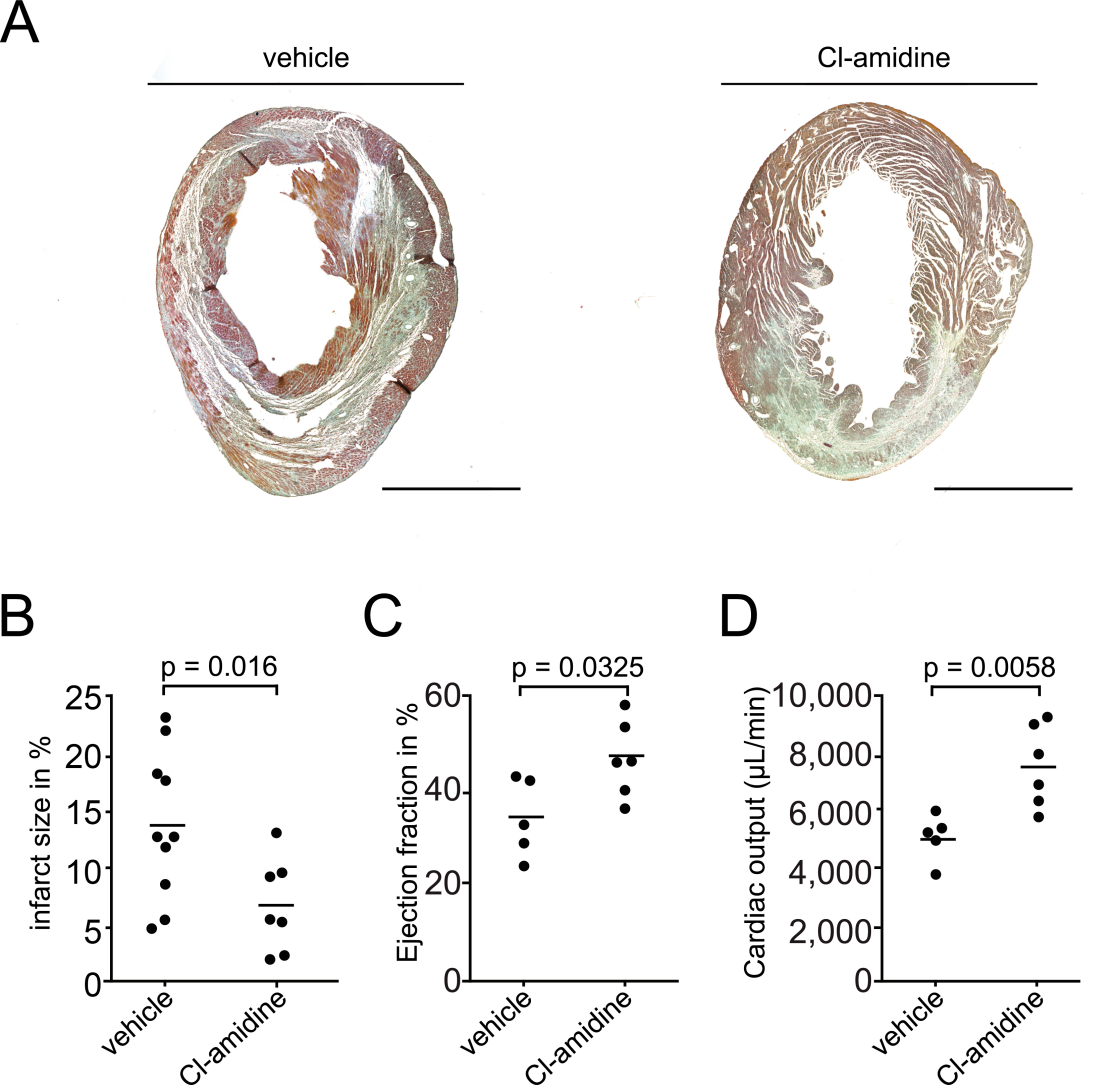
Cl-amidine reduces myocardial ischemia-reperfusion injury. (A) Representative masson-trichrome stainings of myocardial sections from mice 7 days after myocardial ischemia-reperfusion injury treated with vehicle (left) or Cl-amidine (right). Mice treated with Cl-amidine show a decrease in fibrotic tissue compared to vehicle. Bars 2mm. (B) Infarct size 7 days after myocardial ischemia-reperfusion injury in mice treated with vehicle (n = 10) and Cl-amidine (n = 7). (C) Myocardial function was evaluated by measuring ejection fraction (in %) and (D) cardiac output (in μl/min) 7 days after myocardial injury in mice treated with vehicle (n = 5) and Cl-amidine (n = 6). Dots represent individual experiments, lines indicate mean values for each group.

Coronary NET burden has been associated with infarct size in humans [28] and abrogation of NET formation in *Pad4*^*-/-*^ mice reduced infarct size in the early phase of myocardial ischemia [29]. To address whether Cl-amidine reduces myocardial injury and whether these effects persist in the late phase of AMI, we applied Cl-amidine in a model of myocardial ischemia-reperfusion (I/R) injury. In detail, we performed a transient ligation of the LAD for 60 minutes and treated these mice with either Cl-amidine (10 mg/kg) or vehicle. Cl-amidine significantly reduced infarct size after 7 days in comparison to vehicle treated mice (Fig 7A and 7B). This resulted in an improved cardiac function of Cl-amidine treated animals, reflected by an increased ejection fraction (Fig 7C) and higher cardiac output (Fig 7D). Thus, Cl-amidine improves functional outcome after myocardial injury in mice beyond the acute phase of ischemia.

## Discussion

Acute myocardial infarction is a leading cause of death worldwide. Platelets and coagulation pathways are known to critically contribute to arterial thrombosis. However, the role of immune cells and immunomodulatory molecules is less well understood. Therefore, animal models are warranted that produce arterial thrombi closely resembling those in human pathologies such as myocardial infarction. In our present study, we show that the mouse carotid artery injury model based on FeCl_3_ exposure displays many relevant similarities to human coronary thrombi retrieved from patients with AMI. Specifically, the percentage distribution of immune cell populations, the time course of leukocyte accumulation as well as the kinetics of NET formation in this mouse model resembled those of human coronary artery thrombi. Inhibition of NETosis with Cl-amidine is associated with reduced thrombus stability in arterial thrombosis and improved outcome in myocardial infarction. Interestingly, Cl-amidine not only disrupted NETs but also abrogated leukocyte accumulation in arterial thrombi, indicating that the mechanistic effects of this compound extend beyond inhibition of NETosis.

Rupture of an atherosclerotic plaque provides the primary trigger of atherothrombosis in humans resulting in platelet adhesion, activation and aggregation [30]. Apart from platelets, activation of coagulation and subsequent fibrin formation plays a crucial role during thrombus growth and stabilization [2, 30–33]. Immune cells actively contribute to this process [26, 34, 35]. A large body of experimental work in mice has already addressed the mechanisms underlying arterial thrombosis. However, it remained unclear whether these models adequately reflected the situation in humans [9, 10]. We therefore characterized common mouse models of arterial thrombosis applying either chemical (FeCl_3_) or mechanical injury (wire denudation) [11]. Thrombi obtained through wire injury were incompact and did not match the morphology human coronary artery thrombi. Immune cells such as neutrophils were detectable, however, the smaller size and fragmentation of these thrombi precluded an in-depth analysis. We then compared murine arterial thrombi generated in the FeCl_3_ model with human coronary artery thrombi obtained from patients with acute myocardial infarction. We determined whether both pathologies shared key features, such as immune cell composition, NET formation and platelet content. Despite differences in thrombus age and localization of arterial thrombi in mice (3–6 hours, carotid artery) and humans (< 24 hours, coronary artery), we found major analogy between specimens of both species. During the first 12 hours the appearance of leukocytes in both human and mouse thrombi was time dependent, with more immune cells present in older thrombi. In general, we found numerous neutrophils and monocytes in thrombi of mice and humans, a finding quantitatively and qualitatively consistent among both species. Neutrophils thereby represented the most prominent leukocyte subset, which is in line with other recent studies [26, 35], even though human blood consists of a larger neutrophil fraction compared to mouse blood [36]. Mice have approximately three times more platelets in whole blood as compared to humans [37], however, mouse platelets are significantly smaller [38]. It has been speculated that the higher platelet count combined with a smaller volume would result in a similar overall platelet mass in human and mouse blood [39]. In line with this, we detected platelets and fibrinogen/fibrin in comparable amounts in mouse and human thrombi indicating similar contribution to thrombus development.

How do immune cells trigger arterial thrombosis? Recent evidence suggests that the mechanisms used by immune cells to trigger coagulation in nascent arterial thrombi partially mimic those involved in immunothrombosis, a conserved process in which immune cells activate procoagulant pathways to compartmentalize, retain and kill invading pathogens [6]. On the one hand neutrophils can release neutrophil elastase (NE), which stabilizes intraluminal thrombus development by counteracting endogenous anticoagulants (e.g. TF pathway inhibitor) that impede intraluminal coagulation under physiological conditions [40]. In this regard, absence of NE in mice resulted in prolonged bleeding time and reduced thrombus stability [40]. In our comparison of mouse and human specimen, we determined the presence of NE in thrombi of both species suggesting the contribution of neutrophil-derived prothrombotic molecules to arterial thrombosis. A second prominent function of neutrophils is the formation of NETs [41, 42]. These procoagulant DNA matrices are among the key effector molecules of immunothrombosis [8, 43]. NETs contribute to deep vein thrombosis in mice and men [7, 44, 45]. They have also been identified in arterial thrombi of AMI patients, in which NET burden was associated with infarct size [25]. Further, NET formation is enhanced by activated platelets presenting high mobility group box 1 (HMGB1) protein to neutrophils [46]. We show here, that in the mouse carotid artery injury model induced by FeCl_3_, the kinetic of NETs accumulation (i.e. amount of NETs accumulating over time, number of NETs per thrombus area), was similar to that of human patients with AMI. This is an important finding since it will allow future studies in mice studying the consequences of NETosis and targeting NET formation in arterial thrombosis. Further, inhibition of NETosis with the PAD-inhibitor Cl-amidine reduced thrombus stability in the FeCl_3_ model. This resulted in earlier reperfusion of the occluded vessel as observed by intravital microscopy. In line with this, we did not find NETs in thrombi of these mice. Surprisingly, Cl-amidine treatment not only abrogated NET formation but also diminished the number of leukocytes within arterial thrombi. This observation seems to be a local effect on the forming thrombus since leukocyte counts in peripheral blood remained stable, which is in line with the literature [47, 48]. It could be possible that Cl-amidine induces apoptosis in thrombus cells. In fact, it has recently been suggested that PAD inhibition activates the tumor suppressor gene OKL38 thereby inducing apoptosis [49]. The precise mechanism will need to be addressed in more detail in future work. Together, our findings suggest that Cl-amidine not only functions as a potent inhibitor of NET formation but also exerts additional effects on components of the immune system.

NETs can bind effectors of blood coagulation, such as TF and FXII, thereby providing a platform to support their activation [7, 50, 51]. TF initiates the extrinsic pathway of coagulation and leads to fibrin formation after vascular injury [52]. Its binding to NET structures promotes TF exposure at the site of plaque rupture [53]. In this study, Cl-amidine did not affect TF immunofluorescence in arterial thrombi, suggesting that NETs were not essential for exposure of TF in this setting. In fact, various immune cells participate in the storage and exposure of TF, and may contribute to thrombosis [54–56]. However, we observed reduced staining for FXII and fibrinogen in arterial thrombosis of mice treated with Cl-amidine. The initiator protease of the intrinsic pathway of coagulation FXII is activated by binding to negatively charged surfaces such as NETs [7, 40, 57]. Thus, the interaction of NETs could lead to the initiation of the FXII-coagulation pathway thereby triggering fibrin formation, as previously shown for venous thrombosis [7]. Factor XIIa regulates the structure of the fibrin clot independently of thrombin generation through direct interaction with fibrin [58]. Consequently, formation and stabilization of arterial thrombi is reduced in FXII-deficient mice [59]. The data presented here could suggest that the contact pathway-mediated fibrin formation is more important in NET-induced thrombosis. However, our findings are based solely on immunofluorescence analysis. Quantitative data on the protein amount of TF and FXII in the presence and absence of netting neutrophils, as well as measurements of their enzymatic activity, are warranted before such conclusions can be drawn.

As expected from patient cohorts with cardiovascular disease, individuals presenting with AMI were mostly male, old age and presented with several cardiovascular risk factors. We could not identify typical characteristics of patients or thrombi which predisposed for the presence of NETs, however, the study was not designed to specifically assess this question. Another limitation of our study is related to the detection of NETs in human thrombi. NETs were found in only 23% (n = 19) of patient specimen. This could be due to the early, mostly pre-hospital, application of heparin in AMI patients. In fact, the anticoagulant is known for its direct interaction with NETs, causing their degradation both *in vitro* [60] and *in vivo* [7]. Nonetheless the numbers and morphology of NETs in the specimen staining positive for extracellular DNA were similar to that quantified in the mouse FeCl_3_ model.

To test whether treatment with Cl-amidine also conferred a beneficial effect in myocardial infarction beyond the acute phase, we performed an I/R model with transient ligation of the proximal LAD. After 1 week, we found the infarct area to be significantly reduced in the Cl-amidine group, which translated into improved cardiac function. Our data add to previous studies in PAD4-deficient mice showing a reduction of myocardial injury in the early phase (< 24 hours) of infarction [29]. Thus, inhibition of NETosis by Cl-amidine, or potentially other inhibitors [61], may provide a novel treatment strategy in arterial thrombosis. Because this strategy does not seem to affect hemostasis [62], it could provide an improved risk (bleeding)–to–benefit (prevention of ischemic endpoints) profile as compared to conventional antithrombotic therapies.

In conclusion, we have demonstrated that in regard to their cellular composition (i.e. platelets, leukocyte subsets) and prothrombotic molecules (i.e. NETs, fibrin, FXII), FeCl_3_-induced arterial thrombi in mice most closely resemble those of human AMI patients, suggesting that this experimental animal model is more suitable than the one induced by mechanical injury to study the role of immune cells in arterial thrombus development. Further, our study points to yet untargeted molecular pathways and cellular players other than platelets. Current strategies to treat or prevent arterial thrombosis—which consist of a combination of antiplatelet and anticoagulant agents—inhibit major haemostatic pathways and thus share the inherent weakness of affecting haemostasis. Targeting of key molecules in immunothrombosis could help to maximize the efficacy of therapies that prevent arterial thrombosis without increasing the incidence of bleeding complications.

## Supporting information

S1 Fig Platelet accumulation in mouse and human thrombi. (A-B) Immunhistological images of platelet aggregate area in arterial thrombi received from humans (n = 6) and mice (n = 3) after FeCl_3_ injury. Analyses of platelet distribution and thrombus composition showed a comparable morphology. Bars, 100μm. (B) Corresponding quantification of platelet aggregate area. Data are shown as mean ± SD.
(TIF)

S2 Fig Lymphocytes in mouse and human thrombi. (A) Immunohistological images of lymphocytes in arterial thrombi received from humans (n = 6 per group) and mice (n = 3 per group) after FeCl_3_ injury and control stainings. Bars, 50μm. Arrowheads, positive cells. (B) Analysis of lymphocytes shows a comparable small population between patients and FeCl_3_-treated mice. Data are shown as mean ± SD.
(TIF)

S3 Fig Murine arterial thrombosis following infusion of platelets labeled ex vivo. (A-B) Imaging of FeCl_3_-induced arterial thrombosis in mice receiving ex vivo labeled platelets. Isolated platelets were stained with DCF (green) and infused into recipient mice, in which carotid injury was induced. Mice were treated with Cl-amidine or vehicle. (A) Representative intravital microscopy images 5, 10 and 20min after FeCl_3_ injury. Bars, 200μm. (B) Time until occlusion (left) and duration of vessel occlusion (right) after FeCl_3_ exposure. Mice were either untreated (n = 7), or treated with vehicle (n = 9) or Cl-amidine (n = 9). Data are shown as mean ± SD. Results are comparable to experiments in which platelets were directly labeled in vivo (Fig 4).
(TIF)

S4 Fig Comparison of heparin to Cl-amidine in mouse arterial thrombosis. (A) Time until occlusion and duration of vessel occlusion after FeCl_3_ exposure in mice treated with vehicle (n = 8) or Cl-amidine (n = 8) or 100U/kg body weight heparin (n = 3). (B) Quantification of leukocytes in peripheral blood of mice before (n = 5) and 2h after (n = 5) application of Cl-amidine. Data are shown as mean ± SD.
(TIF)

S5 Fig FXII immunofluorescence in human thrombi. Immunohistochemical staining for FXII (green) in human specimen. Human coronary thrombi (top) and liver (bottom row), which serves as positive control. Nuclei were counterstained with Hoechst (including isotype control). Bars, 50μm.
(TIF)
