# Supplementary figures and images for: Histological comparison of arterial thrombi in mice and men and the influence of Cl-amidine on thrombus formation
Source: PLoS One. 2018 Jan 2;13(1):e0190728. doi: 10.1371/journal.pone.0190728 (PMC5749862; doi:10.1371/journal.pone.0190728)

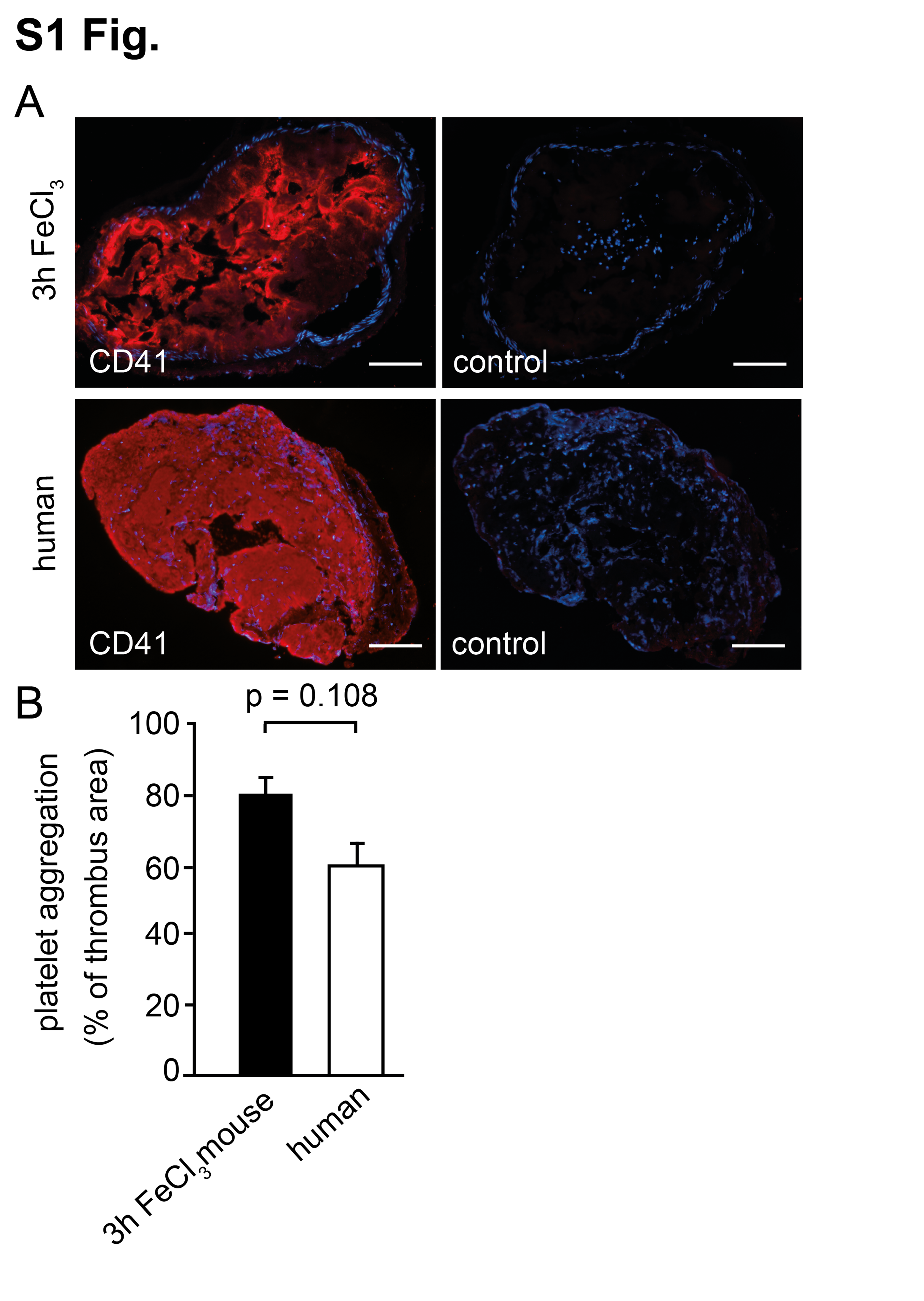

Supplement: S1 Fig — (A-B) Immunhistological images of platelet aggregate area in arterial thrombi received from humans (n = 6) and mice (n = 3) after FeCl3 injury. Analyses of platelet distribution and thrombus composition showed a comparable morphology. Bars, 100μm. (B) Corresponding quantification of platelet aggregate area. Data are shown as mean ± SD. (TIF) [file pone.0190728.s001.tif]

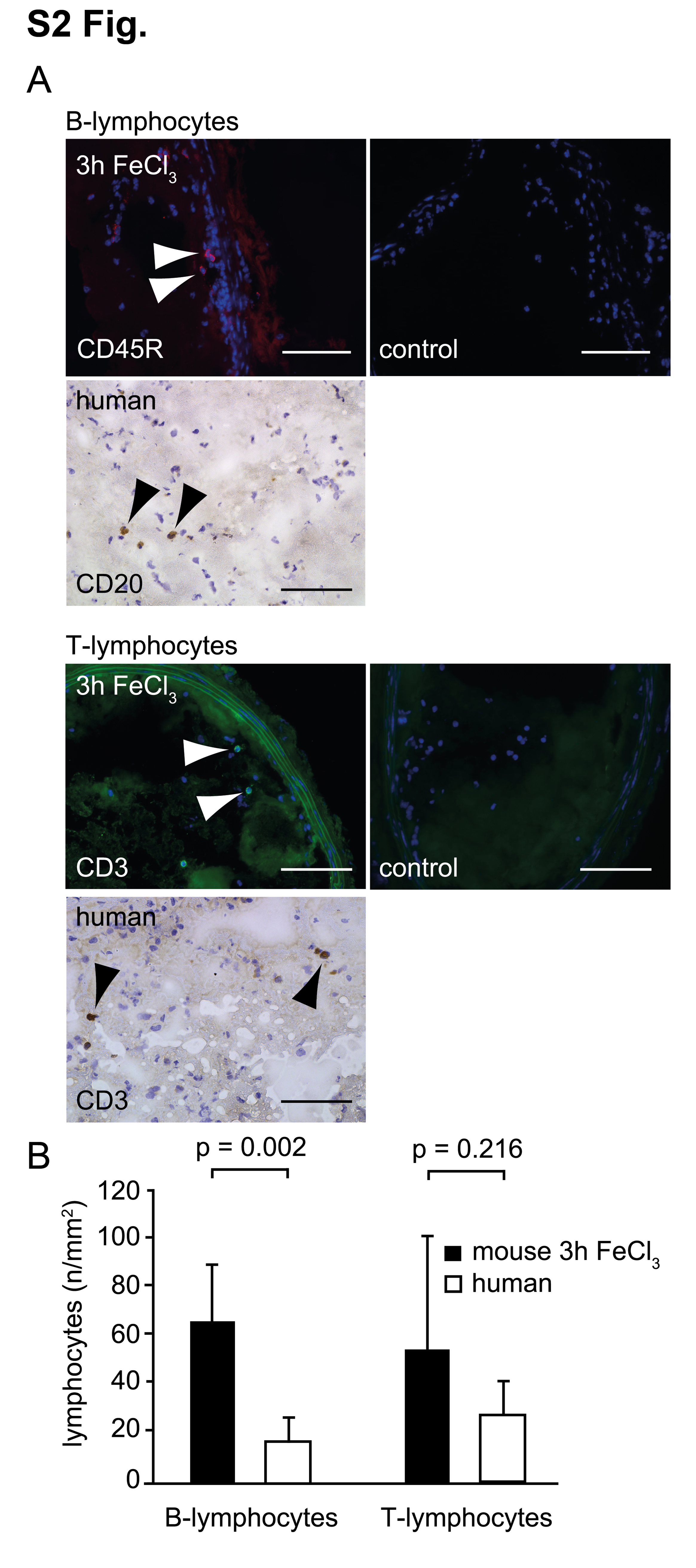

Supplement: S2 Fig — (A) Immunohistological images of lymphocytes in arterial thrombi received from humans (n = 6 per group) and mice (n = 3 per group) after FeCl3 injury and control stainings. Bars, 50μm. Arrowheads, positive cells. (B) Analysis of lymphocytes shows a comparable small population between patients and FeCl3-treated mice. Data are shown as mean ± SD. (TIF) [file pone.0190728.s002.tif]

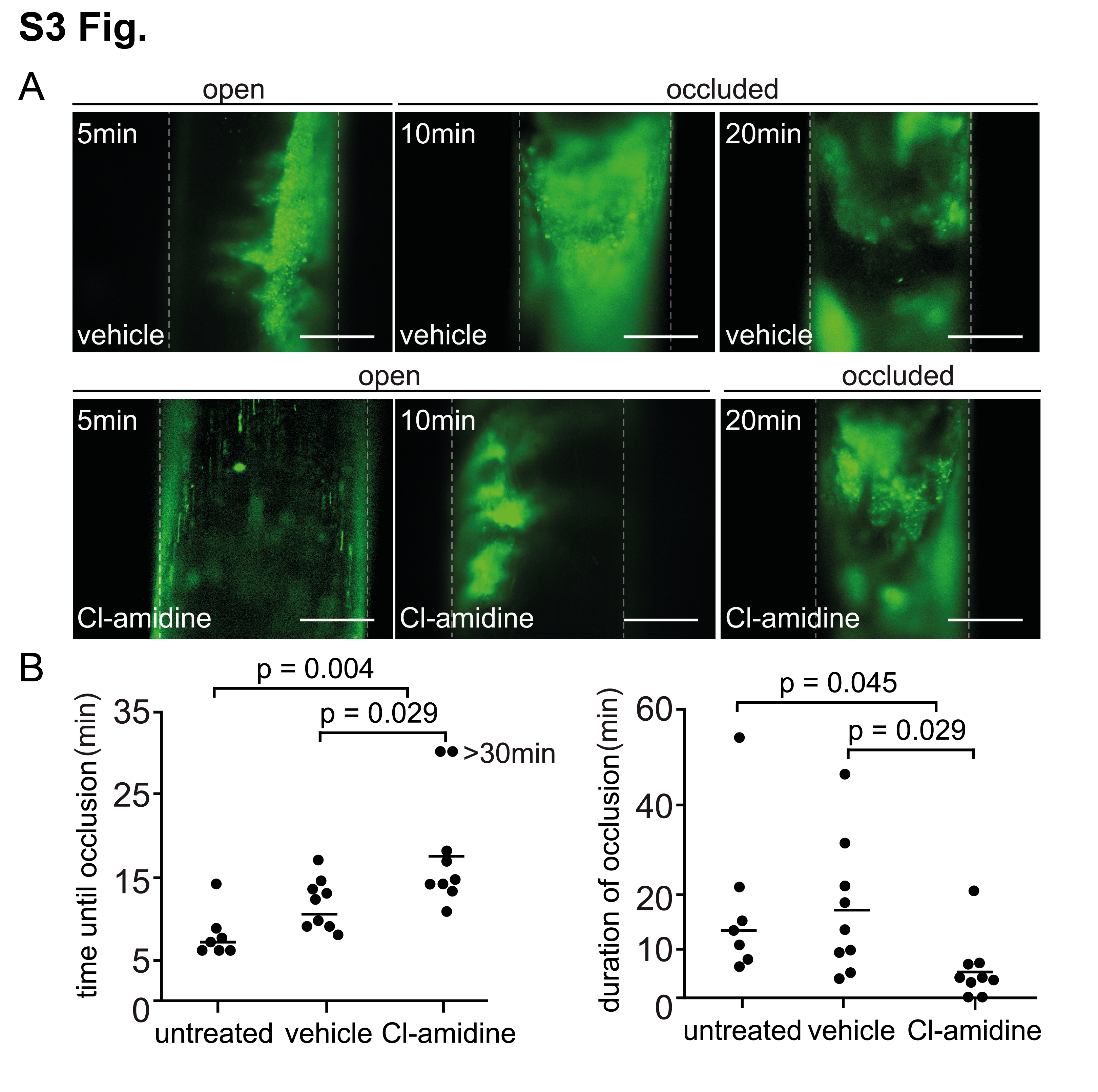

Supplement: S3 Fig — (A-B) Imaging of FeCl3-induced arterial thrombosis in mice receiving ex vivo labeled platelets. Isolated platelets were stained with DCF (green) and infused into recipient mice, in which carotid injury was induced. Mice were treated with Cl-amidine or vehicle. (A) Representative intravital microscopy images 5, 10 and 20min after FeCl3 injury. Bars, 200μm. (B) Time until occlusion (left) and duration of vessel occlusion (right) after FeCl3 exposure. Mice were either untreated (n = 7), or treated with vehicle (n = 9) or Cl-amidine (n = 9). Data are shown as mean ± SD. Results are comparable to experiments in which platelets were directly labeled in vivo (Fig 4). (TIF) [file pone.0190728.s003.tif]

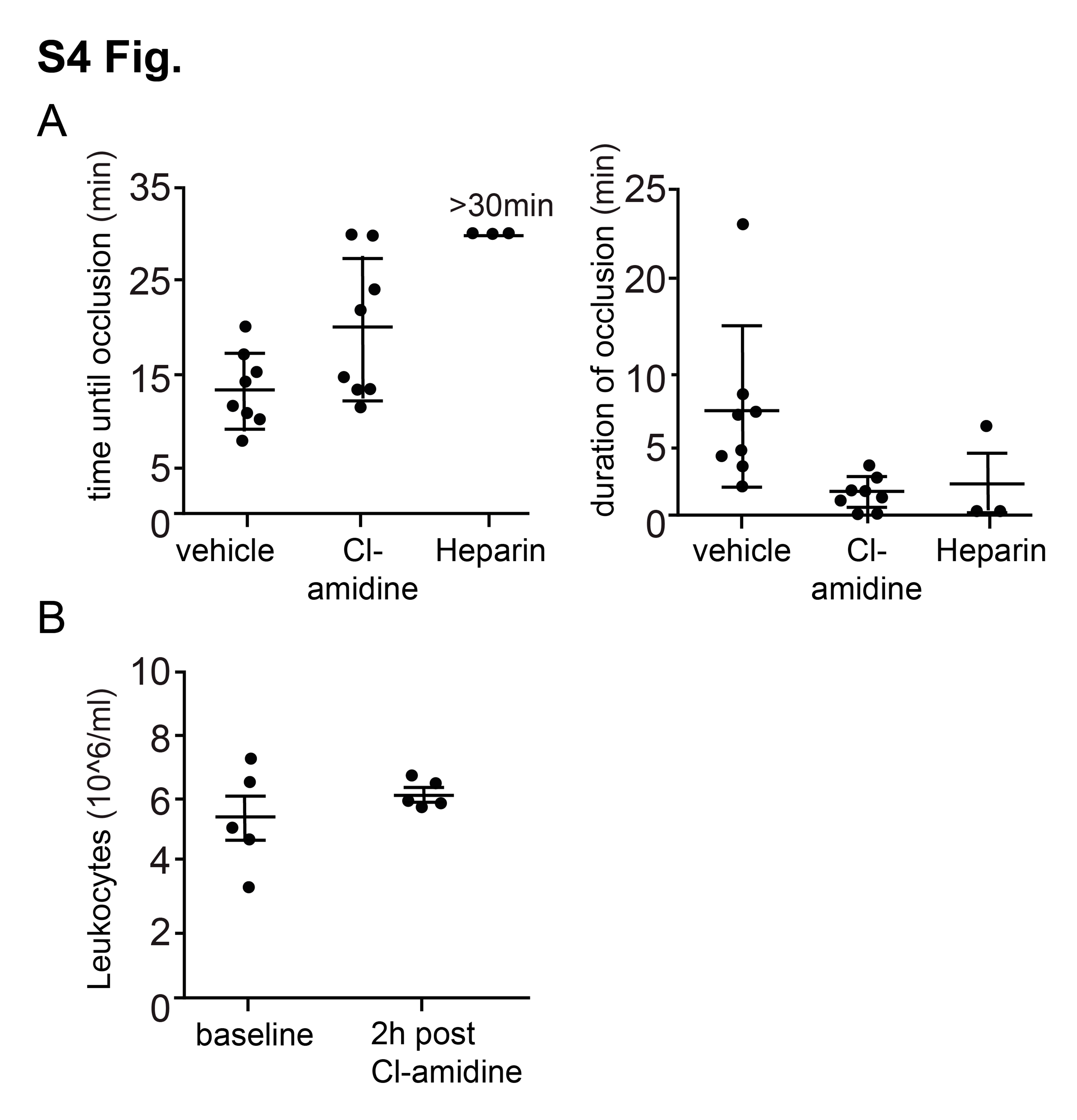

Supplement: S4 Fig — (A) Time until occlusion and duration of vessel occlusion after FeCl3 exposure in mice treated with vehicle (n = 8) or Cl-amidine (n = 8) or 100U/kg body weight heparin (n = 3). (B) Quantification of leukocytes in peripheral blood of mice before (n = 5) and 2h after (n = 5) application of Cl-amidine. Data are shown as mean ± SD. (TIF) [file pone.0190728.s004.tif]

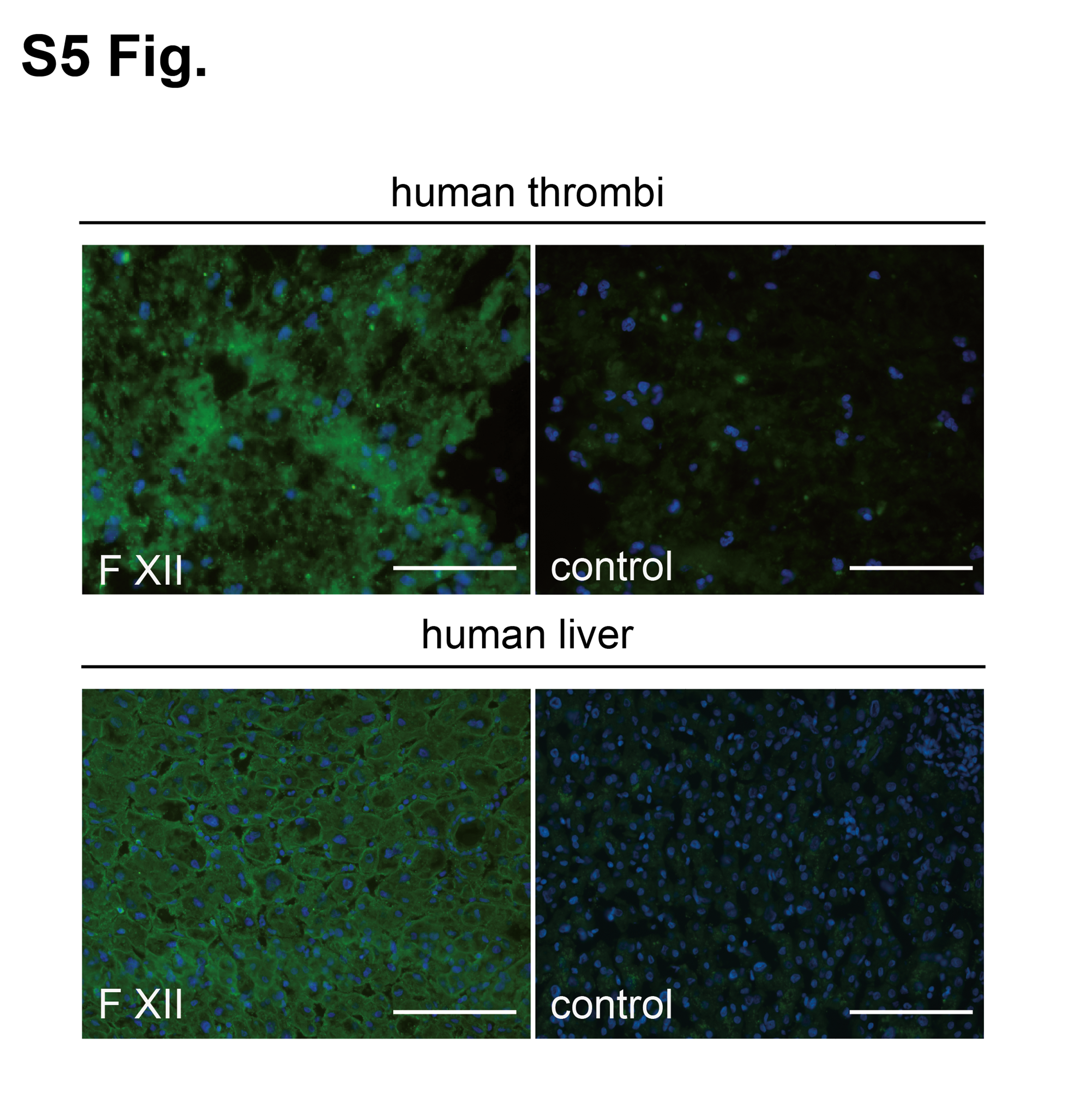

Supplement: S5 Fig — Immunohistochemical staining for FXII (green) in human specimen. Human coronary thrombi (top) and liver (bottom row), which serves as positive control. Nuclei were counterstained with Hoechst (including isotype control). Bars, 50μm. (TIF) [file pone.0190728.s005.tif]
